# *E*-Index for Differentiating Complex Dynamic Traits

**DOI:** 10.1155/2016/5761983

**Published:** 2016-03-15

**Authors:** Jiandong Qi, Jianfeng Sun, Jianxin Wang

**Affiliations:** ^1^School of Information, Beijing Forestry University, Beijing 100083, China; ^2^Center for Computational Biology, Beijing Forestry University, Beijing 100083, China

## Abstract

While it is a daunting challenge in current biology to understand how the underlying network of genes regulates complex dynamic traits, functional mapping, a tool for mapping quantitative trait loci (QTLs) and single nucleotide polymorphisms (SNPs), has been applied in a variety of cases to tackle this challenge. Though useful and powerful, functional mapping performs well only when one or more model parameters are clearly responsible for the developmental trajectory, typically being a logistic curve. Moreover, it does not work when the curves are more complex than that, especially when they are not monotonic. To overcome this inadaptability, we therefore propose a mathematical-biological concept and measurement, *E*-index (earliness-index), which cumulatively measures the earliness degree to which a variable (or a dynamic trait) increases or decreases its value. Theoretical proofs and simulation studies show that *E*-index is more general than functional mapping and can be applied to any complex dynamic traits, including those with logistic curves and those with nonmonotonic curves. Meanwhile, *E*-index vector is proposed as well to capture more subtle differences of developmental patterns.

## 1. Introduction

Whether there are different genes responsible for the formation of a trait and how these genes regulate the trait are of fundamental importance biologically, agriculturally, and/or medically. Quantitative traits, or characteristics varying in degree, can be attributed to the effects of genes and their environment [1]. Lander and Botstein [2] pioneered the systematic integration of molecular genetics and statistical methodologies to dissect quantitative traits to an individual genetic locus, well known as quantitative trait loci (QTLs). Since then, quantitative differences in mass, length, and so forth of the whole individual or an organ in their mature state are used to identify genes [3–8]. According to QTL mapping, individuals with different marker locus genotypes will have different mean values of a quantitative trait, if a QTL is linked to the marker locus.

It should be noted, however, that the single-valued traits are only a portion of the numerous traits, of which many others change with time or other independent variables and are the so-called complex traits. In fact, measurement values in the mature state provide much less information than the growth process leading to it [9]. For example, growth may be defined as quantitative changes in size, mass, or number, and the process is more biologically meaningful than the final state solely: the measurement value of an individual or an organ. The complex traits, which can be expressed as a functional or visually a curve, were thought to be* infinite-dimensional characters* in [10] or* function-valued traits* in [11]. Researchers made effort to study this problem extensively by biological, mathematical, or statistical means [12–15]. And other researchers tried to solve this problem by considering the complex trait (with a set of sampled values) as a bunch of simple traits [16–19]. However, if the values (a set of traits) of a complex trait are considered separately, the relationships between the values are lost or are too time-consuming to capture due to the large size of the residue covariance matrix. But with the eigenvalues of the matrix, the dimensions can be reduced greatly and it becomes feasible for genetic mapping of a large number of traits [20, 21]. These methods, however, do not take into account the developmental mechanisms that regulate trait formation and variation.

Ma et al. [22] proposed* functional mapping*, a statistical framework, for mapping QTL regulating dynamic trajectories of traits. Functional mapping is constructed on the basis of a biological law, as presented by West et al. [23], that the growth of many an organism follows a logistic curve due to the fundamental metabolic principles for allocating energy between maintaining current tissue and gaining biomass. By incorporating the logistic curve, functional mapping differentiates a complex dynamic trait by the parameters of the logistic function, instead of directly by the trait values, and thus makes computation less time-consuming and makes the results more biologically meaningful. Since functional mapping was proposed in [22], there has been a wealth of literature about its variations, improvements, and applications [24–29]. Up to date, functional mapping has successfully applied to associate high dimensions of SNPs with high dimensions of dynamic traits [30].

**Figure 1:**
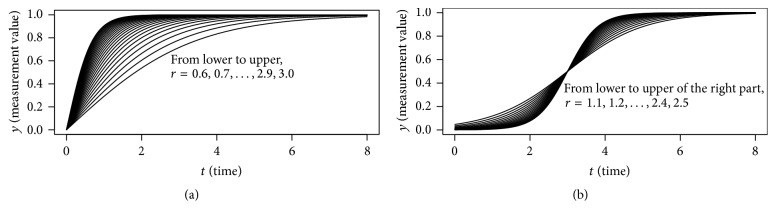
Growth curves with different parameter values. (a) is a bunch of curves described by (1). (b) is a bunch of curves described by (2).

We now briefly review how functional mapping differentiates developmental trajectories. First of all, the growth process of an individual or an organ can be described by a growth curve, a function of a measurable variable against time. Theoretically, a growth curve may provide infinite amount of information, unlike a single measurement value in a mature state. For example, we consider two bunches of growth curves which are described by the following two equations and illustrated in Figure 1:

(1) y=21+e−rt−1,

(2) y=11+e−rt−3.

In practice, discrete values of the developmental process are measured and collected, based on which functional mapping recovers the process by describing it with a curve which is determined by one or more parameters (*r* here indicating the growth rate). We can observe in Figure 1(a) that as the parameter *r* increases, the corresponding growth curve becomes steeper at the beginning part. And in Figure 1(b), a growth curve with greater *r* increases slower at the beginning of the left part and faster at the end of left part. The inclination of the right part is opposite to that of the left part. Therefore the parameter *r* may act as a characteristic value of the bunch to differentiate the curves and thus differentiate growth types or styles.

**Figure 2:**
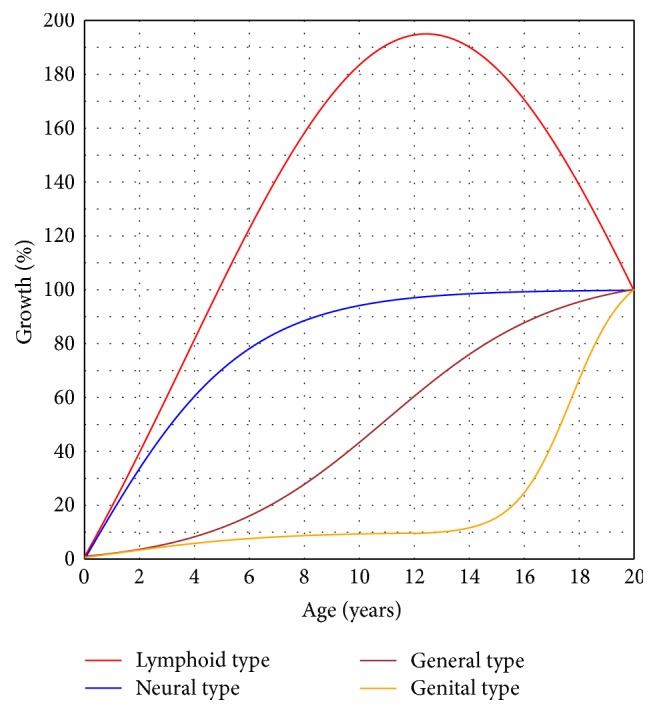
Four major types of growth curves of the organs and the body as a whole, from birth to 20 years [31]. (Later we will obtain their *E*-indices from upper to lower as 1.328, 0.802, 0.459, and 0.191, resp.)

If all growth curves can be described by a function with one or more varying parameters, then we can employ these parameters to be the characteristic values, which is the essence of functional mapping. Unfortunately, no function is qualified for describing all growth types. Specifically, Figure 2 shows Scammon's classic illustration [31] of different growth types of human beings that are almost impossible to describe with a uniform function. Therefore, functional mapping fails to work with curves like the nonmonotonic lymphoid type in Figure 2.

The diversity of growth curves gives rise to a problem: how can we differentiate them with one or more characteristic values? An important characteristic value, *E*-index, will be proposed below and the rest of the paper is organized as follows. *E*-index is defined and its properties are discussed in Section 2. And in Section 3, a statistical framework for *E*-index is given and its effectiveness is validated through simulation studies. *E*-index vector is defined and validated in Section 4. And Section 5 concludes the paper.

## 2. *E*-Index's Definition and Properties

### 2.1. Concept and Definition

As is shown in Figure 1, growth and development may perform faster or slower, earlier or later, due to different types. And the earliness degree of growth that we are to define an index to measure may play an important role to evaluate a growth curve, both mathematically and biologically.

**Figure 3:**
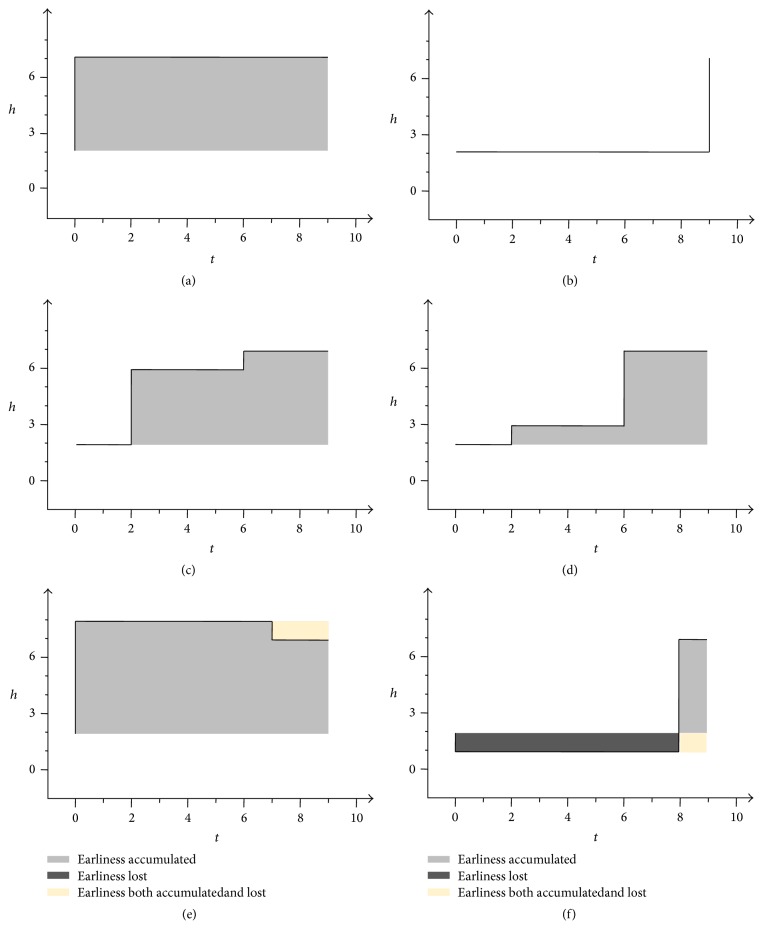
An imaginary scenario about growth. The time interval of the growth is from 0 to 9, and the measurement values are all 2 at the beginning point and are all 7 at the end point.

It is common sense that a growth curve is continuous and smooth, but in order to elucidate the concept of *E*-index intuitively and for simplicity, we design an imaginary scenario in which an individual gains part of its height instantaneously (though this is impossible), as shown in Figure 3. It takes each of the 6 individuals indicated in Figure 3 exactly 9 units of time (from 0 to 9) to gain 5 units of height (from 2 at the beginning to 7 at the end).

Take in Figure 3(c), for instance, first. The individual indicated by it keeps its original height 2 for the first 2 units of time, and then its height instantaneously increases by 4 units at the time point 2. After that, it keeps the height for 4 units of time, until it increases its height again by 1 unit at time point 6. Finally it keeps the height 7 to the end point.

Compared with (c), the individual indicated by (d) grows “later” since, at the “earlier” time point 2, it increases less, while, at the “later” time point 6, it increases more. Intuitively, the earliness degree of the individual indicated by (c) is more than that of the individual by (c). But we need to quantitatively measure the earliness degree to systematically reflect the difference and comparison. Obviously, two factors are to be considered: increased height and the time span from the time point when increasement occurs to the end time point, and therefore we use their product to represent the earliness degree.

On the basis of the analysis and discussion above, we are now able to calculate the earliness degree of individual (c) as the sum of the areas of two rectangles, one being 4 (increased height) by 7 (time span to the end) and the other being 1 (increased height) by 3 (time span). The area sum is 4 × 7 + 3 × 1 = 31, which can be standardized to be 31/45 = 0.689, by being divided by the area of the entire rectangle of 5 (total increased height) by 9 (whole time span from beginning to end).

Similarly, the earliness degree of (d) is calculated as (1 × 7 + 4 × 3)/45 = 0.422, which is much less than 0.689, the earliness degree of (c).

Using the same method we can calculate the earliness degree of the other 4 individuals, with those of (a) and (b) being trivially 1 and 0, respectively. But the cases of (e) and (f) are more complicated, since the height of (e), before reaching the end time point, has increased to a value 8, a greater value than that of the mature state, 7; and the height of (f) has decreased to a value 1, a smaller value than the beginning value 2. Nevertheless, the earliness degree of (e) can be calculated as (6 × 9 − 1 × 2)/45 = 1.156, a value greater than 1, and that of (f) as (−1 × 9 + 1 × 6)/45 = −0.067, a negative value.

We denote the quantitative earliness degree as *E*-index for short. Though imaginary and impossible in real life, the scenario presented in Figure 3 gives us a helpful intuition and clue to define the *E*-index rigidly.

To give the definition, we do not require a growth curve to be globally differentiable, but it is currently required to be piecewise differentiable, which as we will see later is not necessarily met. And we hence give the definition of *E*-index.

**Definition 1 .**

Suppose that the growth curve is a continuous function *f*(*t*) defined on a closed interval [*a*, *b*], *a* < *b*. {*p*_*i*_}_*i*=0_^*n*^ is a sequence of points, *p*_0_ = *a*, *p*_*n*_ = *b*, 0 ≤ *i* < *j* ≤ *n*. Also suppose that *f*(*t*) is differentiable on the open interval (*p*_*i*−1_, *p*_*i*_), *i* = 1,2,…, *n*, and that *f*′(*t*) is its derivative function. Then we define the *E-index* of the growth curve as follows:

(3) $$\begin{array}{c} E_{a}^{b}\left(\;f\;\right)=\frac{1}{\left(b-a\right)\left(f\left(b\right)-f\left(a\right)\right)}\overset{n-1}{\underset{i=1}{\sum }}\overset{b}{\underset{a}{\int }}f^{\prime }\left(\;t\;\right)\left(\;b-t\;\right)dt. \end{array}$$

It should be noted that *f*′(*t*), the growth rate at time point *t*, is undefined on each inner split point. But this would not change the integration result, even if we set the growth rate at such point to be any value. For simplicity, we denote *E*_*a*_^*b*^(*f*) as *E*(*f*).

How early or how late the growth rate *f*′(*t*) occurs is our key concern for growth, and the expression *f*′(*t*)(*b* − *t*) in (3) quantifies the degree of earliness. The greater the product value *f*′(*t*)(*b* − *t*) is, the earlier the growth or development occurs. In this sense, the *E*-index measures how early growth occurs in the whole process by accumulating the product along the time.

### 2.2. Properties of *E*-Index

From the definition above, we can derive several of *E*-index's properties which are to be discussed in the form of propositions. However, their proofs are all omitted since they can be found in calculus textbooks or related literature.

**Proposition 2 .**

If the growth curve function *f*(*t*) defined on the closed interval [*a*, *b*] is strictly monotonically increasing and is globally differentiable, then its *E*-index can be calculated with the following equation:

(4) Ef=1b−afb−fa∫abftdt−fab−a=∫abftdtb−afb−fa−fafb−fa.

Proposition 2 provides us an alternative approach to calculate the *E*-index and reveals to us the relations between the integrations along horizontal direction and along vertical direction.

**Proposition 3 .**

The conclusion of Proposition 2 still holds if the growth curve function is still globally differentiable, but not necessarily monotonic.

**Figure 4:**
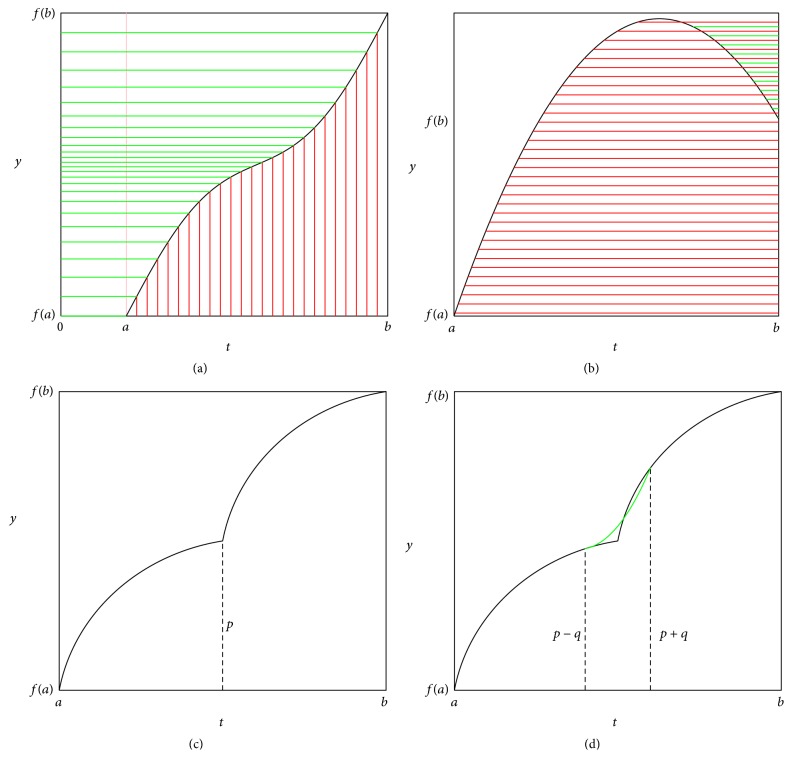
Illustrations for the proposition proofs. (a) is a growth curve strictly monotonically increasing. (b) is a nonmonotonic growth curve. (c) is a piecewise smooth growth curve. (d) is a resulting growth curve by smoothening that in (c) near the unsmooth internal point.

We can illustrate the proof with Figure 4. Suppose there is only one inner extreme point (we can prove it similarly with more inner extreme points). The integration for the left part of the curve forms the red area, while that for the right part forms the green area which is negative. And their sum is the area between the curve and the horizontal line *y* = *f*(*a*), which is the conclusion we wanted.

**Proposition 4 .**

The conclusion of Proposition 3 still holds if the growth curve function is piecewise differentiable.

Propositions 2–4 indicate that we can calculate *E*-index with (4), no matter whether the growth curve is monotonic or not and no matter whether it is piecewise smooth or globally smooth. In fact, if the growth curve function is not differentiable, even not continuous, we are still able to calculate its *E*-index with (4), without changing the meaning of *E*-index.

Typically, the measurement value in the growth process is between the value at the beginning and that at the end. And we have still another proposition for this situation.

**Proposition 5 .**

If the growth curve function *f*(*t*) is defined on [*a*, *b*] and *f*(*a*) ≤ *f*(*t*) ≤ *f*(*b*), then 0 ≤ *E*(*f*) ≤ 1.

But *E*(*f*) may get a value greater than 1 if, for some *t*, *f*(*t*) is greater than *f*(*b*) or even be a negative value if, for some *t*, *f*(*t*) is less than *f*(*a*).

Proposition 5 dictates the range of *E*-index, and we can design a growth curve function whose *E*-index is any designated value in the range.

## 3. Validating *E*-Index's Effectiveness

*E*-index can be easily calculated with integration operation stated in (4). However, is it as effective as the function parameters, say, *r* in (1) and (2), to differentiate growth curves? Or in addition, is it able to differentiate the growth curves without a uniform function in Figure 2? Growth curves are usually formed by collecting successive measurements and finding a function (sometimes difficult to find) approximately fitting the data. But is *E*-index applicable in this situation? We will answer all these questions in the following subsections.

### 3.1. Contrasting *E*-Index and Function Parameters

**Figure 5:**
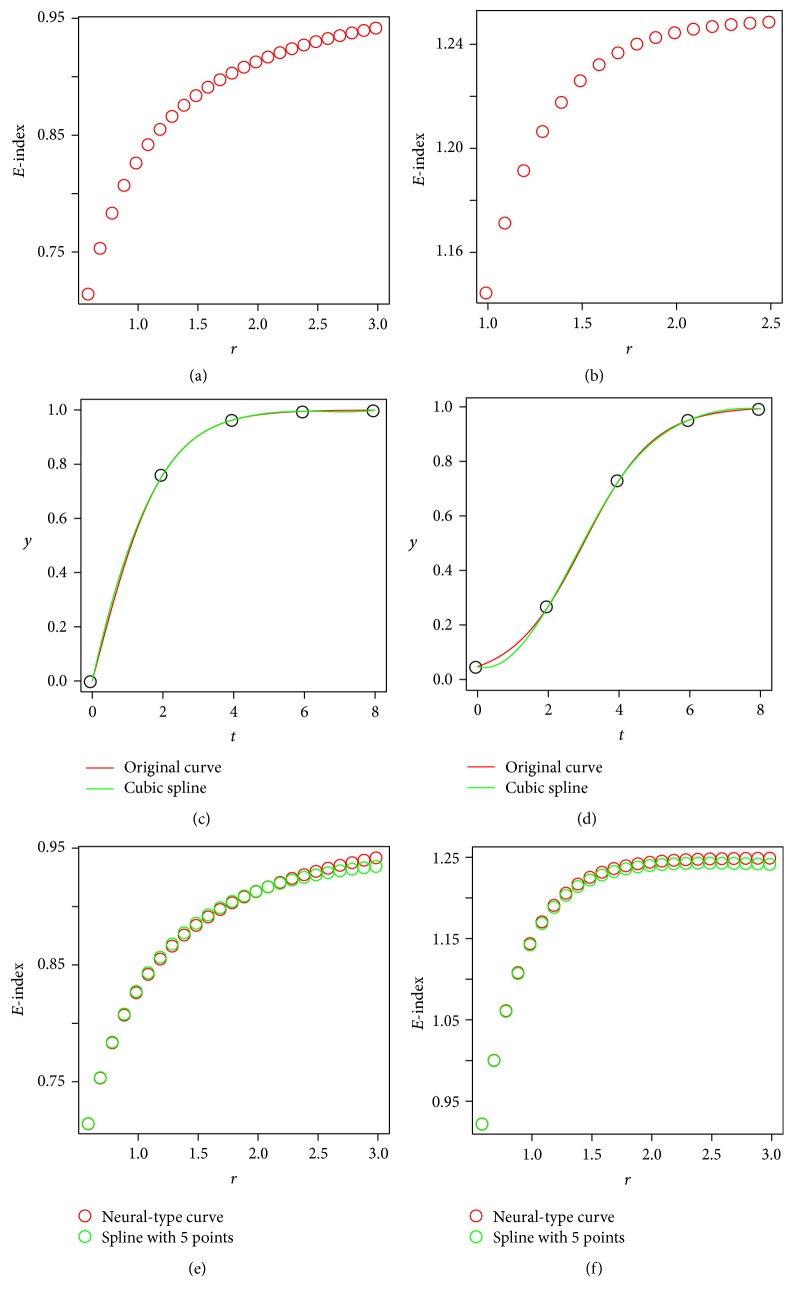
Simulations validating the effectiveness of *E*-index. (a) is *E*-indices of growth curves in Figure 1(a) plotted against parameter *r*. (b) is *E*-indices of growth curves in Figure 1(b) plotted against parameter *r*. (c) contrasts a growth curve in Figure 1(a) with *r* = 1 and its spline. (d) contrasts a growth curve in Figure 1(b) with *r* = 1 and its spline. (e) compares *E*-index values of the bunch of functions in Figure 1(a) and those of their splines. (f) compares *E*-index values of the bunch of functions in Figure 1(b) and those of their splines.

The function parameter *r* in the 2 bunches of growth curves can differentiate the curves, as is illustrated in Figure 1. We are trying to find out whether *E*-index is capable of doing so, and the results are shown in Figure 5.

For each of the 25 values of *r* in Figure 1(a), the *E*-index of the corresponding growth curve function is calculated with (4) as follows:

(5) $$\begin{array}{c} E\left(\;r\;\right)=E\left(\;f_{r}\left(\;\cdot \;\right)\;\right)=\frac{1}{8}\overset{8}{\underset{0}{\int }}\left(\;\frac{2}{1-e^{-rt}}\;\right)-1. \end{array}$$

The relation between the values of the parameter *r* and the corresponding *E*-index values is plotted in Figure 5(a). It can be observed that as *r* increases, the *E*-index value increases accordingly, which implies that *E*-index is capable of differentiating growth curves as the parameter *r* that is related to growth rate. For the bunch of growth curves in Figure 1(b), we can obtain similar result illustrated in Figure 5(b).

### 3.2. *E*-Index Applied in Nonuniform Functions

In some cases, we may use *E*-index as an equivalent of function parameters, to differentiate growth curves of a uniform type. In addition, we may continue to apply *E*-index to differentiate them without uniform function describing them. For instance, the 4 types of growth curves in Figure 2 are lymphoid, neural, general, and genital, respectively. Specifically, we can use the following 4 functions to precisely describe them:

(6) fLymphoidt=810e−t−17.432/153.1175−110.5,0≤t≤20,fNeuralt=2001+e−0.35t−100,0≤t≤20,fGeneralt=1081+e−0.32t−11−2.1,0≤t≤20,fGenitalt=161+e−0.35t−1−6,0≤t≤12,1001+e−t−17.7+9.2,12<t≤20.

Applying (4) once again to the above functions, we will obtain the corresponding *E*-indices as 1.328, 0.802, 0.459, and 0.191 for the lymphoid, neural, general, and genital type of growth curves, respectively. This result is consistent with our observation and intuition: growth and development occur earliest for the lymphoid type, comparing to the other three types of growth curves; and the genitals grow and develop latest among the four types.

This example illustrated that *E*-index may, at least in some cases, differentiate growth curves even without uniform function describing them.

### 3.3. *E*-Index of Spline Interpolation

In order to differentiate growth curves by function parameters, we have to assume the function type first and then calculate parameters making the function fit the successively collected measurement values best. The resulting parameters do not work well if the function does not fit the data well.

In fact, spline interpolation performs well to find a smooth function piecewise defined by polynomials. Unfortunately, splines are not uniform functions and therefore, function parameters do not work either for the case of splines. *E*-index, however, does work in this situation. Based on the successively collected measurements, we can define a smooth function to fit the data by spline interpolation and then calculate the *E*-index of the function. The resulting *E*-indices will provide help to differentiate the corresponding growth indicated by the collected measurements.

We will consider 2 growth curves. The first one is described by (1) with the parameter *r* = 1. Suppose that we do not have any knowledge of the curve type and all that we have is the function values of 5 interpolation points evenly dispersed in the time domain. A typical kind of spline, cubic spline, is calculated and compared to the original curve in Figure 5(c). The second growth curve is described by (2) with *r* = 1, and the derived cubic spline and the original curve are contrasted in Figure 5(d).

It is observed from Figures 5(c) and 5(d) that the spline fits the original growth curve well (and will fit it better with more interpolation points), which indicates that *E*-index works well even without knowledge of the growth curve type.

Next, spline is calculated for each of the functions in the bunch illustrated in Figure 1(a). For the same value of the parameter *r*, *E*-index of the original function and that of the spline are calculated, respectively. And the obtained *E*-index values are contrasted in Figure 5(e). Similarly, the *E*-index values are contrasted in Figure 5(f) for the original functions illustrated in Figure 1(b) and their splines. The results are encouraging, since *E*-index values of the splines are quite close to those of the original functions if the number of inner interpolation points is 5 or more (see Figures 5(e) and 5(f)).

### 3.4. Statistical Framework of *E*-Index

How can we apply *E*-index to differentiate complex dynamic traits? We are typically given two genotypes *A* and *B* with *m* samples of *A* and *n* samples of *B*, each sample measured at *T* time points. And our purpose is to judge whether the genotypes significantly affect the phenotypes.

Suppose that the value vector of the *i*th sample of *A* is *v*_*i*_^*A*^ = (*v*_*i*,1_^*A*^, *v*_*i*,2_^*A*^,…, *v*_*i*,*T*_^*A*^), *i* = 1,2,…, *m*, and that of the *j*th sample of *B* is *v*_*j*_^*B*^ = (*v*_*j*,1_^*B*^, *v*_*j*,2_^*B*^,…, *v*_*j*,*T*_^*B*^), *j* = 1,2,…, *n*. The computation steps are as follows, using *t*-test of (*m* + *n* − 2) degrees of freedom to discover the significance.

(a) For each *v*_*i*_^*A*^ and *v*_*j*_^*B*^, we can use spline interpolation to get continuous functions (curves) *f*_*i*_^*A*^(*t*) and *f*_*j*_^*B*^(*t*).
(b) Then (4) is employed to calculate each *E*-index of the curves, namely, *E*_*i*_^*A*^ and *E*_*j*_^*B*^, respectively, for *i* = 1,2,…, *m* and *j* = 1,2,…, *n*. And we denote that *E* ^*A*^ = (*E*_1_^*A*^, *E*_2_^*A*^,…, *E*_*m*_^*A*^) and *E* ^*B*^ = (*E*_1_^*B*^, *E*_2_^*B*^,…, *E*_*n*_^*B*^).
(c) After that we define a test statistic,
  (7) $$\begin{array}{c} t=\frac{\bar{E_{A}}-\bar{E_{B}}}{\sqrt{s^{2}\left(1/m+1/n\right)}}, \end{array}$$
  where $\bar{E_{A}}$ is the mean of the vector *E* ^*A*^ and $\bar{E_{B}}$ is the mean of the vector *E* ^*B*^ (here we suppose that $\bar{E_{A}}>\bar{E_{B}}$) and the common variance
  (8) $$\begin{array}{c} s^{2}=\frac{\left(m-1\right)s_{A}^{2}+\left(n-1\right)s_{B}^{2}}{m+n-2}, \end{array}$$
  where *s*_*A*_^2^ is the sample variance of the vector *E* ^*A*^ and *s*_*B*_^2^ is that of *E* ^*B*^.
(d) We can test the null hypothesis that the two groups of samples are not significantly different:
  (9) $$\begin{array}{c} H_{0}\text{:}\begin{array}{c} \bar{E_{A}}-\bar{E_{B}} \end{array}=0, \end{array}$$
  versus the alternative hypothesis that the two groups are significantly different:
  (10) $$\begin{array}{c} H_{1}\text{:}\begin{array}{c} \bar{E_{A}}-\bar{E_{B}} \end{array}>0. \end{array}$$
  *H*_0_ will be rejected if
  (11) $$\begin{array}{c} t>t_{\alpha }\left(\;m+n-2\;\right); \end{array}$$
  otherwise *H*_0_ will be accepted, where *t* is the computation result of (7) and *t*_*α*_(*m* + *n* − 2) is the *t*-distribution value with the confidence level *α* and (*m* + *n* − 2) degrees of freedom.

### 3.5. Applying *E*-Index

With the statistical framework of *E*-index in the previous subsection, we can now apply it to differentiate complex dynamic traits.

**Figure 6:**
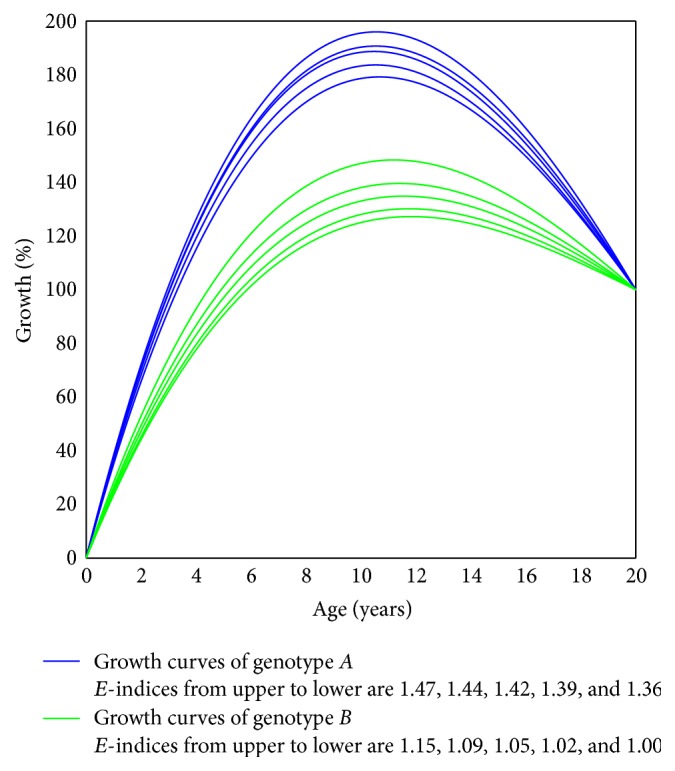
Simulated organ growth curves of different genotypes *A* and *B* with sample size 5 each.

Two bunches of growth curves of genotypes *A* and *B*, respectively, are generated by simulation, and they, together with their *E*-indices, are illustrated in Figure 6.

The relative measurement value of each sample at the beginning time point 0 is 0 percent, and that at the ending time point 20 is 100 percent. Consequently, we are not able to differentiate them merely by the measurement value at the mature state and have to resort to the difference of developmental processes.

Intuitively, the two groups are far apart. But we fail to apply the functional mapping framework to differentiate the two groups, since there exist no parameters like *r* in (1) and (2) responsible for the curve shape. In addition, each curve is nonmonotonic, which functional mapping is not able to deal with.

But using the statistical framework given in Section 3.4, we get the standard deviations of the *E*-indices for genotypes *A* and *B*,  *s*_*A*_ = 0.0428 and *s*_*B*_ = 0.0597, respectively. We have by (8) the common sample variance *s* ^2^ = 0.0027 and by (7) the test statistic *t* = 10.77, much larger than *t*_0.01_(5 + 5 − 2) = 2.896, which means that there is sufficient evidence to indicate that the genotypes are clearly responsible for the developmental processes of the organs.

## 4. *E*-Index Vector

As is mentioned earlier, a curve may theoretically provide infinite amount of information about growth. Though the *E*-index is sometimes capable of differentiating growth curves, it is after all only one characteristic value revealing one aspect of information. Therefore, it is natural for us to extend *E*-index into *E*-index vector.

### 4.1. Definition of *E*-Index Vector

**Figure 7:**
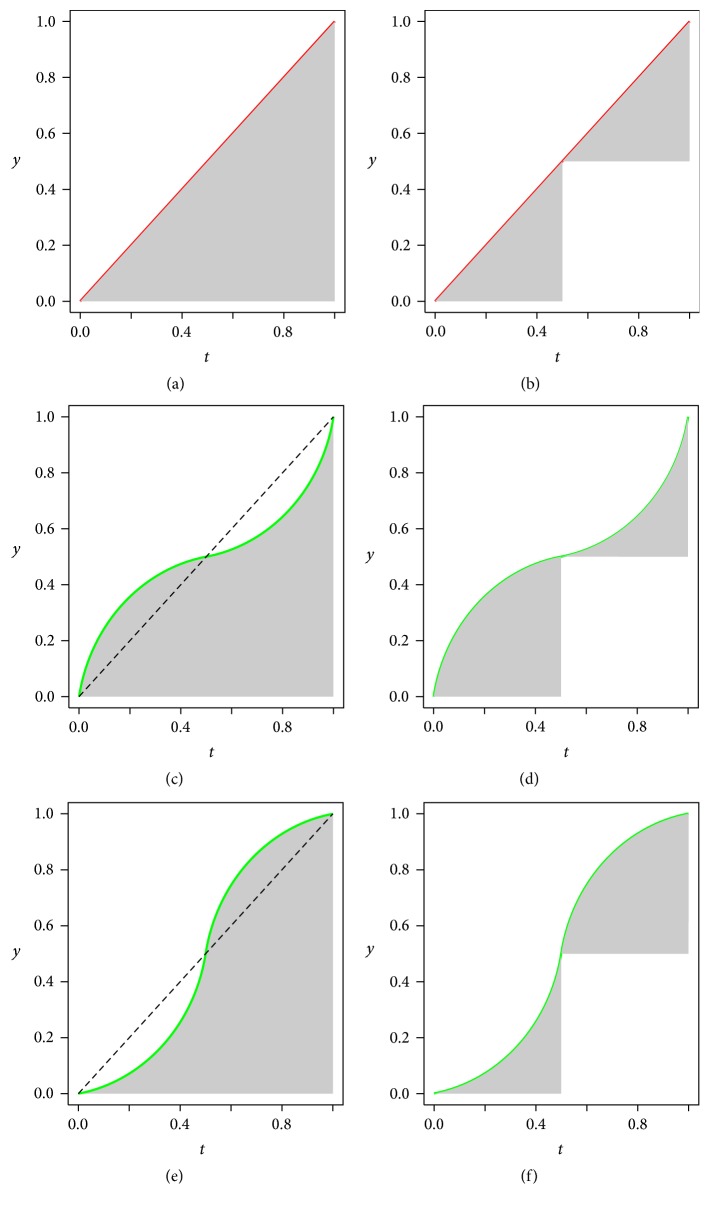
The limitation of *E*-index and the definition of *E*-index vector. (a) and (b) illustrate *E*-index and *E*-index vector for constant growth rate, respectively. (c) and (d) illustrate *E*-index and *E*-index vector for higher growth rate in earlier half and lower growth rate in later half. (e) and (f) illustrate *E*-index and *E*-index vector for lower growth rate in earlier half and higher growth rate in later half. *E*-indices of the curves in (a), (c), and (e) are of the same value 1. *E*-index vectors for the curves in (b), (d), and (e) are (0.5, 0.5), (0.716, 0.284), and (0.284, 0.716), respectively.

Where and why is *E*-index insufficient to differentiate growth curves? An example from Figures 7(a), 7(c), and 7(e) will illustrate this.

Comparing the sizes of shade in Figures 7(a), 7(c), and 7(e), we will find that 3 totally different growth curves lead to the same *E*-index value (0.5). This is mainly due to the fact that the effect caused by the higher growth rate in Figure 7(b) or Figure 7(c) is counteracted by lower growth rate earlier or later.

This example indicates that *E*-index does not work in some cases to differentiate growth curves. Consequently, we have to move forward for a more sophisticated tool. Naturally we will extend *E*-index into *E*-index vector.

**Definition 6 .**

Suppose that *f*(*t*) describing a growth curve is continuous on a closed interval [*a*, *b*], *a* < *b*. And suppose that *Q* = {*p*_*i*_}_*i*=0_^*n*^ is a prescribed sequence of points, *p*_0_ = *a*, *p*_*n*_ = *b*, *p*_*i*_ < *p*_*j*_, 0 ≤ *i* < *j* ≤ *n*. Then the *E-index vector* of *f*(*t*) according to *Q* is defined as follows:

(12) $$\begin{array}{c} V_{Q}\left(\;f\;\right)=\left(\;nE_{p_{0}}^{p_{1}}\left(\;f\;\right),nE_{p_{1}}^{p_{2}}\left(\;f\;\right),\ldots ,nE_{p_{n-1}}^{p_{n}}\left(\;f\;\right)\;\right). \end{array}$$

*V*_*Q*_(*f*) is denoted as *V*(*f*) for simplicity and (*nE*_*p*_0__^*p*_1_^(*f*), *nE*_*p*_1__^*p*_2_^(*f*),…, *nE*_*p*_*n*−1__^*p*_*n*_^(*f*)) as (*E*_1_(*f*), *E*_2_(*f*),…, *E*_*n*_(*f*)).

The *E*-index vectors of the growth curves in Figures 7(b), 7(d), and 7(f) are calculated with (12). And the three resulting *E*-index vectors, (0.5, 0.5), (0.716, 0.284), and (0.284, 0.716), are apparently different, as is also illustrated with shaded areas in Figure 7. But how can we evaluate this difference quantitatively? In order to answer this question, we above all give another definition as the following.

**Definition 7 .**

Suppose *f*(*t*) and *g*(*t*) are two functions defined on a closed interval, describing two growth curves with the same prescribed sequence of points *Q* = {*p*_*i*_}_*i*=0_^*n*^. Then the* growth dissimilarity* between *f*(*t*) and *g*(*t*) is defined as follows:

(13) $$\begin{array}{c} D\left(\;f,g\;\right)={\left[\;\sum_{i=1}^{n}{\left(\;E_{i}\left(\;f\;\right)-E_{i}\left(\;g\;\right)\;\right)}^{2}\;\right]}^{1/2}. \end{array}$$

It is easy to prove that growth dissimilarity satisfies distance axioms; that is,

(14) Df,f=0,Df,g=Dg,f,Df,g+Dg,h≥Df,h.

And we hence have transformed problems about growth curves into problems about vectors which will help to analyze the relation between different growth curves in Figure 7. Denote the functions describing the growth curves in Figures 7(b), 7(d), and 7(f) as *f*_1_(*t*), *f*_2_(*t*), and *f*_3_(*t*), respectively. According to (13), their dissimilarities are calculated and listed as follows:

(15) Df1,f2=0.355,Df1,f3=0.355,Df2,f3=0.611.

The results above show that, in the growth perspective of earlier and later halves, *f*_2_(*t*) is more similar to *f*_1_(*t*) than it is to *f*_3_(*t*), which is consistent with what is observed in Figure 7.

Different weights can be designated to differently important phases of growth according to specific problems. So the growth dissimilarity defined in (13) can be accordingly redefined as the following equation with *W*_*i*_ denoted as the *i*th weight:

(16) $$\begin{array}{c} D\left(\;f,g\;\right)={\left[\;\sum_{i=1}^{n}W_{i}{\left(\;V_{i}\left(\;f\;\right)-V_{i}\left(\;g\;\right)\;\right)}^{2}\;\right]}^{1/2}. \end{array}$$

Equation (16) enables *E*-index vector to help differentiate two growth curves. What is more important, however, is to differentiate a set of growth curves or to divide them into groups or clusters, which will be discussed in the next subsection.

### 4.2. Grouping or Clustering Growth Curves by *E*-Index Vector

More and more growth traits are available and they can be described by growth curves. Studies [12] indicate that growth traits are powerful to identify genes some of which cannot be identified by traits only in one time point.

In order to identify genes with growth traits, we are required to divide into groups all growth curves that are as similar as possible in the same group while being as dissimilar as possible in different groups. But a common situation we are encountering is that it is difficult for us to obtain reasonable groups.

*E*-index, *E*-index vector, and the growth dissimilarity definition based on these two concepts may help us to group or cluster the growth curves.

With the *E*-index vectors, we can define describing rules for a curve group. Take the growth curves in Figures 7(b), 7(d), and 7(e), for instance. We define the rule describing the first group as “the first component of the *E*-index vector is greater than 0.6 and the second less than 0.4.” And the second rule is defined as “the first component is less than 0.6 and the second greater 0.4.” These two rules describe and define two groups, with growth curve (d) in the first group and growth curves (b) and (e) in the second one.

Though describing rules are capable of grouping growth curves, human experts are involved in prescribing the rules, and thus the rules, consequently the grouping results, may be different from person to person.

Unlike the grouping technique with describing rules, the clustering technique, *k*-mean algorithm, and its variations are almost automatic. It is a recursive algorithm with *k* randomly selected centers. To cluster the growth curves, the growth dissimilarities between each growth curve and each center are calculated with (16), and a group corresponding to a center will include all the growth curves nearer to its center than to the other centers. This process continues until the inclusion of each group keeps unchanged.

**Figure 8:**
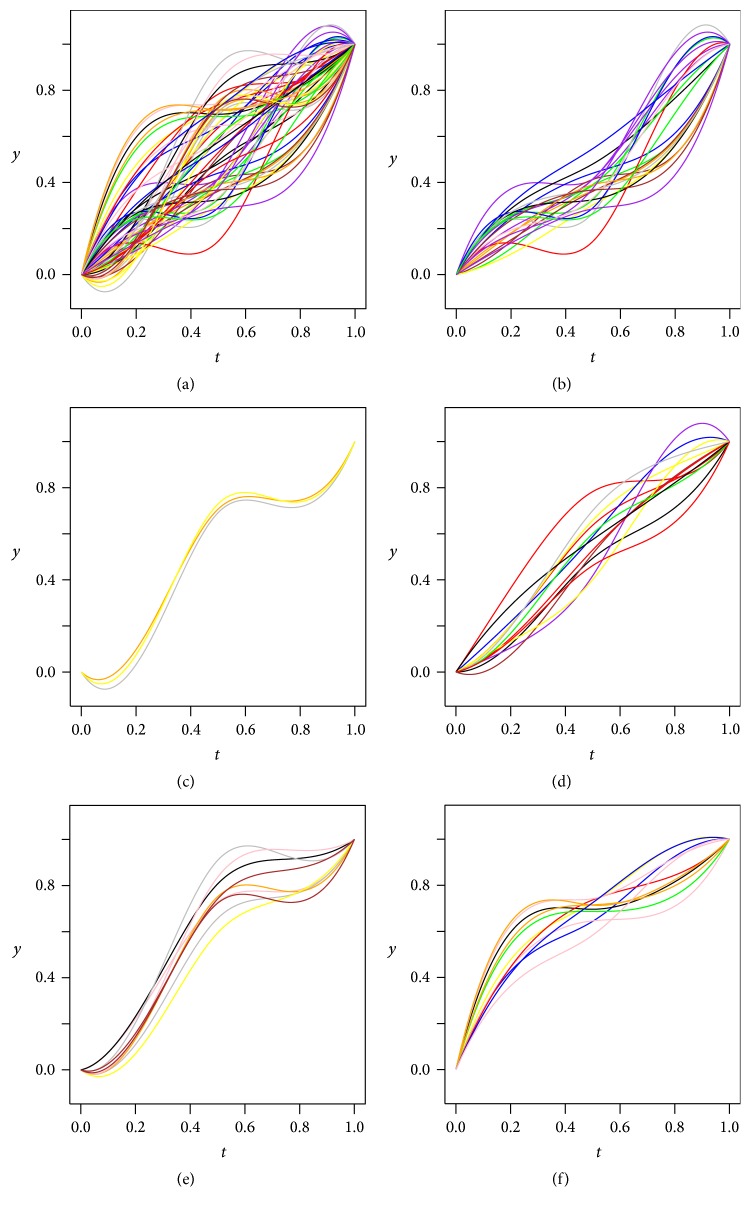
Clustering growth curves based on their *E*-index vectors. (a) illustrates 60 randomly generated growth curves, with a random color designated to each of them. (b)~(f) are the resulting groups after applying *k*-mean algorithm, with a group in each figure. Each growth curve in (b)~(f) retains its shape, position, and color from its original in (a).

We simulated the *k*-mean algorithm by randomly generating 60 growth curves and dividing them into 5 clusters by the algorithm, according to *E*-index vector definition in (12) and growth dissimilarity definition in (16). In Figure 8, the primitive growth curves and the resulting groups are displayed. It can be seen from Figure 8 that each group represents a distinct growth style.

## 5. Conclusions

In order to generalize functional mapping and overcome the shortages of it, *E*-index and *E*-index vector are proposed in this paper, respectively, by means of measuring earliness degree of growth or development in the overall process and in a growth phase. We summarize their features as follows.

(i) *E*-index is capable of differentiating growth curves (such as logistic curves) as function parameters are in the applications of functional mapping. Like functional mapping, *E*-index is good at differentiating growth trajectories with the same values of mature state, which traditional QTL takes as the same. In this sense, *E*-index generalized functional mapping.
(ii) *E*-index is sometimes unavailable according to its primitive definition given in Definition 1, due to strict restrictions. But it is always available for a growth curve and easier to calculate from another perspective stated in Proposition 4. Moreover, measuring the earliness degree of growth or development, *E*-index is as biologically meaningful as the important curve parameters employed in functional mapping.
(iii) A function globally and thoroughly describing the process of growth is unnecessary for calculating *E*-index. In fact, a cubic spline (as employed in [32]) approximates that well. Furthermore, *E*-index can be applied in any period of the developmental process; on the contrary, functional mapping can only be applied to the whole process in order to get suitable parameters.
(iv) Being a key and general characteristic value though, *E*-index provides limited information. As an extension of *E*-index, *E*-index vector is focused on the growth in different phases, the number of which may vary, and the time spans for the same vector may not be of equal length, according to the application background and requirements.
(v) By extracting the growth information in a curve and forming a vector, we can use well developed techniques for analysis, such as describing rules and *k*-mean algorithm.
(vi) *E*-index vector helps us reveal detailed characteristics in growth curves. It may be looked on as a microscope employed to observe a desired level of growth detail.
